# The Role of Microglia in the Spread of Tau: Relevance for Tauopathies

**DOI:** 10.3389/fncel.2018.00172

**Published:** 2018-07-10

**Authors:** Juan R. Perea, María Llorens-Martín, Jesús Ávila, Marta Bolós

**Affiliations:** ^1^Department of Molecular Neuropathology, Centro de Biología Molecular “Severo Ochoa”, CBMSO, CSIC, Madrid, Spain; ^2^Network Center for Biomedical Research on Neurodegenerative Diseases (CIBERNED), Madrid, Spain; ^3^Department of Molecular Biology, Faculty of Sciences, Universidad Autónoma de Madrid, Madrid, Spain

**Keywords:** microglia, tauopathies, Alzheimer’s disease, tau protein, phagocytosis

## Abstract

Tauopathies are neurodegenerative diseases which course with the accumulation of Tau, mainly in neurons. In addition, Tau accumulates in a hyperphosphorylated and aggregated form. This protein is released into the extracellular space and spreads following a stereotypical pattern, inducing the development of the disease through connected regions of the brain. Microglia—the macrophages of the brain—are involved in maintaining brain homeostasis. They perform a variety of functions related to the surveillance and clearance of pathological proteins, among other dead cells and debris, from the extracellular space that could compromise brain equilibrium. This review focuses on the role played by microglia in tauopathies, specifically in Alzheimer’s disease (AD), and how the uncoupling of activation/phagocytosis functions can have fatal consequences leading to the development of the pathology.

## Introduction

Microglia are the macrophages of the brain that are in charge of maintaining central nervous system (CNS) homeostasis. These cells are constantly surveying their microenvironment and, in response to small variations, they rapidly change their morphology, become activated, secrete pro- and anti-inflammatory molecules, and phagocytose foreign material and other potentially toxic elements that can alter the equilibrium of the brain (Ransohoff and El Khoury, 2016; Wolf et al., 2017).

Some neurodegenerative diseases known as tauopathies, such as Alzheimer’s disease (AD), course with the abnormal intracellular accumulation of pathological Tau species (Ballatore et al., 2007; Goedert and Spillantini, 2011; Arendt et al., 2016). This toxic Tau is released outside the cells (Simón et al., 2012; Pooler et al., 2013), and in the extracellular space, it can interact with other cells (Gómez-Ramos et al., 2006), be internalized (Wu et al., 2013; Bolós et al., 2015), and trigger cell to cell Tau spread (Medina and Avila, 2014a; Calafate et al., 2015). Therefore, in order to protect healthy cells, microglia should activate mechanisms to remove this extracellular pathogenic protein and, by doing so, halt the progression of the disease. However, in some pathological situations, the uncoupling of the activation/phagocytosis functions of microglia can accelerate the progression of neurodegeneration (Sierra et al., 2013; Bolós et al., 2017a; Leyns and Holtzman, 2017).

## Physiological and Pathological Tau Species

First discovered by Weingarten et al. (1975), Tau is a microtubule-associated protein present in the CNS. The human Tau gene, *Mapt*, is situated on chromosome 17q21 and comprises 16 exons (Pittman et al., 2006). Some of these exons in the CNS, namely 1, 4, 5, 7, 9, 11, 12 and 13, are constitutive, while the others, 2, 3 and 10, are subjected to alternative splicing (Andreadis, 2005). The latter process generates six isoforms of Tau in the adult human brain that differ in the number of N-terminal insertions, tubulin binding domains and microtubule-binding repeats (Goedert and Spillantini, 2011). Alternative splicing of exon 10 is highly regulated through a complex mechanism. The deregulation of this process contributes to neurodegeneration (Liu and Gong, 2008).

Tau is largely found in axons (Aronov et al., 2001). It binds to microtubules and regulates the dynamics of these structures, thus allowing the reorganization of the cytoskeleton and axonal transport. However, Tau is also localized to the somatodendritic compartment, where it plays key roles at synapses (Drubin and Kirschner, 1986; Ittner and Götz, 2011). The expression of Tau protein, as well as its function, are complexly regulated by alternative splicing and several post-transcriptional modifications, including phosphorylation, glycosylation, acetylation and truncation, among others (Morris et al., 2015; Avila et al., 2016).

Tauopathies are characterized by the accumulation of aggregated and/or hyperphosphorylated Tau (Ballatore et al., 2007; Holtzman et al., 2011; Medina et al., 2016). Tauopathies are a group of neurological disorders that include corticobasal degeneration (CBD), frontotemporal dementia (FTD) and Parkinsonism linked to chromosome 17 (FTDP-17), progressive supranuclear palsy (PSP), Pick’s disease (PiD), chronic traumatic encephalopathy (CTE), argyrophilic grain disease (AGD) and AD, the latter being the most common tauopathy (Spillantini and Goedert, 2013; Arendt et al., 2016). In tauopathies, Tau which is a highly soluble hydrophilic protein, detaches from microtubules and accumulates, forming intracellular hyperphosphorylated aggregates or inclusions, such as the neurofibrillary tangles (NFTs) found in AD brains (Brion et al., 1985; Wood et al., 1986; Wischik et al., 1988; Zhang et al., 2009). These structures damage cell function and produce neuronal cell death and neurodegeneration.

## Spread of Tau

Current hypotheses postulate that Tau is released from its intracellular localization into the extracellular space, from where it spreads following a stereotypic pattern, from the transentorhinal cortex to the hippocampus and, finally, to the neocortex (de Calignon et al., 2012; Holmes and Diamond, 2014; Hyman, 2014; Brettschneider et al., 2015; Hu et al., 2016). The spread of Tau through connected areas of the brain contributes to cognitive decline in age-associated tauopathy. It has been proposed that Tau is released by dying neurons, and, in addition, that it can be secreted physiologically (Pooler et al., 2013; Yamada et al., 2014; Kanmert et al., 2015; Pérez et al., 2016). Indeed, its physiological release was postulated as a mechanism to prevent the toxicity caused by elevated intracellular Tau (Karch et al., 2012; Simón et al., 2012; Pooler et al., 2013).

Extracellular Tau can interact with neighboring cells, such as neurons or glia (Gómez-Ramos et al., 2006; Bolós et al., 2015; Luo et al., 2015), and cause severe damage (Bolós et al., 2017b). The spread of Tau from adjacent cells has been proposed to be one of the mechanisms underlying the progression of tauopathies (Medina and Avila, 2014b; Mirbaha et al., 2015). Tau propagation between synaptically connected neurons have been reported by many groups (de Calignon et al., 2012; Yamada et al., 2014; Brettschneider et al., 2015); however, other spread mechanisms may occur. In this regard, we and others have recently demonstrated that extracellular soluble Tau, which encompasses small oligomers of Tau, interacts with microglia and is internalized by these cells (Luo et al., 2015; Sanchez-Mejias et al., 2016; Bolós et al., 2017a). Therefore, the dysfunction of microglia can also contribute to the spread of Tau (Maphis et al., 2015). Remarkably, augmented levels of exosome-associated Tau have been reported in the cerebrospinal fluid and blood of individuals with AD and FTD (Saman et al., 2012; Fiandaca et al., 2015). These findings suggest that the exosomal process is involved in the cell to cell spread of Tau (Aguzzi and Rajendran, 2009; Rajendran et al., 2014; Polanco et al., 2016). Furthermore, the inhibition of exosome biosynthesis and depletion of microglia halt the spread of this protein in a mouse model of tauopathy (Asai et al., 2015). These studies highlight the involvement of microglia, through phagocytosis and the release of Tau-containing exosomes, in the propagation of Tau pathology.

## Microglia—Key Cells Maintaining Brain Homeostasis

Microglia are the resident immune cells of the CNS and represent approximately 15% of the cells in this area (Lawson et al., 1990; Perry, 1998; Alliot et al., 1999). They were termed by Pío del Río Hortega. He hypothesized that microglia have the ability to rapidly change their morphology, proliferate and migrate in response to changes in the microenvironment. He proposed that the basic function of these cells was phagocytosis. During early embryonic stages, microglia originate from myeloid progenitors in the yolk sac that migrate into the brain before the blood-brain barrier is formed (Alliot et al., 1999; Ginhoux et al., 2010). Under physiological conditions, microglia proliferate and self-renew constantly without a contribution from bone marrow-derived macrophages in order to maintain cell numbers. In contrast, in the presence of a disease, circulating peripheral monocytes contribute to the microglia population maintenance (Ginhoux et al., 2010; Bruttger et al., 2015).

Microglia are highly dynamic cells that regulate several processes during both development and adulthood in the CNS (Ransohoff and El Khoury, 2016). They perform a wide variety of functions, including the following: constant surveillance; removal of pathogens; phagocytosis of apoptotic cells and cellular debris; secretion of growth factors, pro-inflammatory and anti-inflammatory signals; synapse remodeling and elimination, among others (Sierra et al., 2010, 2014; Paolicelli et al., 2014; Michell-Robinson et al., 2015; Wolf et al., 2017). The main distinctive of microglia is their capability to quickly alter their morphology and function in response to variations in their microenvironment (Town et al., 2005; Karperien et al., 2013). In this regard, there are two main types of microglia, namely resting (ramified) and activated (ameboid; Town et al., 2005). Many studies have tried to explain the correlation between these two types of microglia and their roles in physiological and pathological conditions, but the exact function of the different morphological states remains unknown. Activated microglia are often classified into inflammatory (M1) and alternatively activated (M2) phenotypes (Boche et al., 2013; Tang and Le, 2016). M1 microglia produce and release pro-inflammatory cytokines, such as tumor necrosis factor (TNF)-α, interleukin (IL)-6, IL-12, IL-1β, IL-23, nitric oxide (NO), among others (Hanisch, 2002). In addition, it has been described that M1 microglia predominate at the site of injury (Loane and Byrnes, 2010). In contrast, M2 microglia express extracellular matrix molecules and anti-inflammatory molecules, such as transforming growth factor (TGF)-β and IL-10, and express phagocytic activity. M2 microglia act later, at a period more associated to repair processes (Michell-Robinson et al., 2015). However, this M1 and M2 classification is oversimplified since mixed activation phenotypes can co-occur (Ransohoff, 2016a).

Surveillance microglia participate in CNS homeostasis by actively contacting surrounding cells (Nimmerjahn et al., 2005). In this regard, soluble and membrane-bound cytokines mediate neuron-microglia communication. This cross-talk manages the balance between the neuroprotective and harmful actions of microglia on neurons (Sierra et al., 2014; Wolf et al., 2017). One example is how neurons modulate microglial function through the CX3CL1/CX3CR1 axis (Harrison et al., 1998; Biber et al., 2007). As CX3CL1 is produced almost exclusively by neurons (Kim et al., 2011) and the receptor CX3CR1 is expressed almost exclusively on the surface of microglia (Zhan et al., 2014), this axis is a sensor of the neuronal signals. It has been recently shown that suitable cross-talk between microglia and neurons is essential for the accurate functional maturation of newborn granule neurons in the hippocampus and has an important role in the regulation of emotional behavior (Bolós et al., 2018). In this regard, it has been demonstrated that Tau binds to CX3CR1 increasing its own internalization by microglia, and that Tau competes with CX3CL1 to bind to this receptor (Bolós et al., 2017a). In addition, it has been shown that there is less internalization of phospho-Tau by microglial CX3CR1 than for the non-phosphorylated form of the protein. These observations thus suggest that phospho-Tau is internalized through other mechanisms. Furthermore, an increase in the expression of the CX3CL1/CX3CR1 axis and a greater number of activated microglia in the hippocampus of patients with an advanced stage of AD has been shown. However, the higher amount of phospho-Tau observed in brain tissue of AD patients should not be automatically interpreted to be the result of increased Tau phagocytosis, since this process may be compromised at some point of the disease development. Actually, a reduction in the phagocytic activity of microglia was proposed since a decrease in the number of phagocytic pouches in these cells was observed. These data support the idea that the disconnection of microglial activation and phagocytosis happens at advanced stages of AD.

Other examples of dynamic communication between microglia and neurons that could be relevant in AD include the following neuron/microglia cross-talk: CD200/CD200R; ATP/P2Y; P2X; and CD22/CD45 (Mott et al., 2004; Minas and Liversidge, 2006; Surprenant and North, 2009; Verkhratsky et al., 2009; Eyo and Wu, 2013); among others.

In addition to performing active surveillance, microglia are tissue-resident macrophages, meaning that they are the phagocytic cells of the brain. Their function encompasses the clearance of cellular material and debris in order to maintain brain homeostasis. Phagocytosis is modulated in terms of activation or inhibition through various mechanisms (Parnaik et al., 2000; Peri and Nüsslein-Volhard, 2008; Sierra et al., 2010; Paolicelli et al., 2011; Schafer et al., 2012). One such mechanism is exerted by Toll-like receptors (TLRs), which recognize pathogens and induce a phagocytic response by microglia (Ribes et al., 2009). In addition, apoptotic neurons are phagocytosed after been recognized by various receptor systems like those described above. Other multiple factors regulate phagocytosis, such as the ciliary neurotrophic factor, CNTF, glia-derived neurotrophic factor, GDNF and macrophage colony-stimulating factor, M-CSF, the latter potentiating the phagocytic capacity of microglia (Mitrasinovic and Murphy, 2003; Chang et al., 2006; Lee et al., 2009). Substrate-bound complement component C1q enhances both FcR- and CR1-mediated phagocytosis (Webster et al., 2000), whereas the prostanoid receptor subtype 2, EP2, downregulates phagocytosis (Shie et al., 2005). Therefore, the dysregulation of microglial phagocytosis mechanisms can impair the clearance of debris, thus inducing or exacerbating the inflammatory response and causing brain diseases such as AD.

## Microglia Dysfunction Contributes to Neurodegeneration

**Figure 1:**
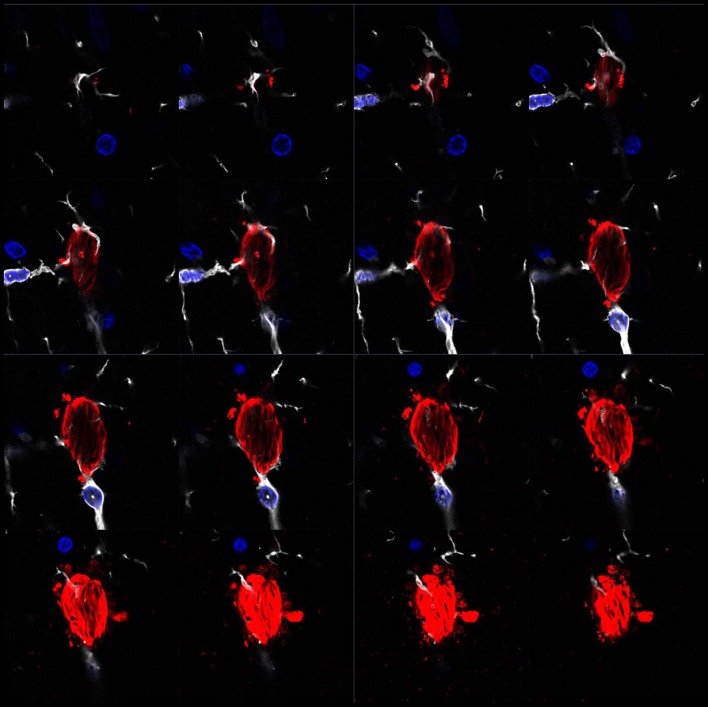
Microglia surrounding phospho-Tau in human brain tissue. Separate z-projection images of a z-stack image showing phospho-Tau surrounded by microglia published by our group previously (Bolós et al., 2015). The authors have the appropriate permissions from the copyright holders. Images show microglia in white (Iba-1), phospho-Tau in red (S396) and nuclei in blue (DAPI).

Genome-wide association studies (GWAS) have recently identified several risk genes for AD. Specifically, the triggering receptor expressed on myeloid cells 2, TREM2 and cluster of differentiation 33 (CD33) can lead to a reduction in the activity of the complement system and decreased phagocytosis (Bradshaw et al., 2013; Jonsson et al., 2013). Several lines of evidence suggest that microglia prevent AD by stimulating the clearance of Aβ. Numerous microglial receptors appear to play a pivotal role in the clearance of this peptide (Yu and Ye, 2015), including microglial scavenger receptor 1 (Scara-1), cluster of differentiation 36 (CD36), the receptor for the Fc region of IgG IIb (FcγRIIb) and RAGE, the receptor of advanced glycation end products. For instance, during the progression of AD, the expression of these receptors in microglia decreases, and these cells lose their ability to clear Aβ (Derecki et al., 2014). The increase in the number of studies addressing the clearance of Aβ by microglia in recent years has brought about a deeper understanding of the contribution of microglia to clearance of this peptide. However, the clearance of extracellular Tau by microglia has received less attention. Nevertheless, several studies coincide on the crucial role played by microglial phagocytosis in the clearance of Tau and, consequently, in avoiding the spread of Tau and the progression of AD (Luo et al., 2015; Bolós et al., 2017c; Leyns and Holtzman, 2017; Figure 1). Therefore, it is now necessary to channel our efforts into elucidating the mechanisms by which pathological forms of Tau are cleared by glial cells to maintain brain homeostasis.

Abnormal and continuous activation of microglia has been reported in AD (Town et al., 2005; Ransohoff and El Khoury, 2016; Bolós et al., 2017c). In this regard, our recent data support the notion that although microglia are more activated in advanced stages of AD, their capacity to phagocytose Tau is impaired, thereby suggesting that there is a disconnection of microglial activation and phagocytosis at later stages of the disease (Bolós et al., 2017a). This effect has already been described for Aβ clearance by microglia in AD (Xiang et al., 2016) and in other neurodegenerative diseases (Sierra et al., 2013). Indeed, it has been proposed that neuron/microglial crosstalk is compromised in chronic neurodegenerative conditions (Sheridan and Murphy, 2013; Simon et al., 2018), and this impairment may explain the chronic maintenance of a pro-inflammatory state in AD.

The transcriptome of microglia has been studied by several groups (Chiu et al., 2013; Hickman et al., 2013; Srinivasan et al., 2016; Keren-Shaul et al., 2017; Mathys et al., 2017). The overall gene expression profile of microglia revealed the upregulation of genes involved in neuroprotection and host defense during adulthood and aging in healthy brains (Hickman et al., 2013). However, using single-cell analysis in AD and other models of neurodegenerative disease, such as lateral amyotrophic sclerosis, the authors observed changes in several genes involved in microglia functions as the disease progressed (Chiu et al., 2013; Mathys et al., 2017). In a very interesting study using single-cell RNA-seq in an AD model, Keren-Shaul et al. (2017) identified a novel microglia subtype associated with neurodegenerative diseases (DAM) that is activated in a two-step process. In early stages, the expression of some genes, such as *Cx3cr1*, is upregulated, while other genes, such as *Trem2*, are downregulated. In late stages of AD, this ratio changes inversely, meaning that *Trem2* is activated and *Cx3cr1*, among other genes, is downregulated. The observation that the expression of this set of genes is associated with phagocytic activity provides additional evidence of impaired microglia elementary functions in neurodegenerative diseases.

**Figure 2:**
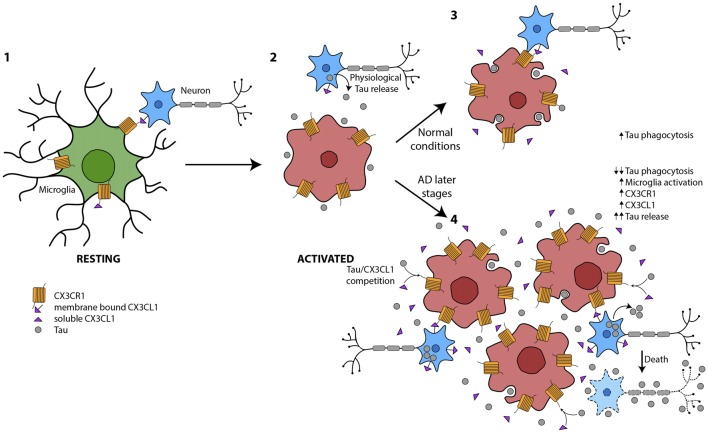
Global picture of the contribution of microglia to Tau propagation. (1) Microglia in resting state where membrane-bound CX3CL1 and soluble isoforms are binding to the microglial CX3CR1. The CX3CL1/CX3CR1 axis is functioning properly. (2) Under several circumstances, such as the presence of Tau in the extracellular space, among others, microglia are activated. (3) Tau is phagocytosed by microglia under normal conditions. There is an equilibrium between microglial activation and phagocytosis in part mediated by CX3CL1/CX3CR1. (4) At later stages of Alzheimer’s disease (AD), there is an increase in Tau in the extracellular space, probably as a result of high neuron mortality. This Tau competes with CX3CL1 for binding to CX3CR1. Therefore, the phagocytosis of Tau is impaired and microglia are more activated and proliferate. The amount of CX3CL1 and CX3CR1 increase. However, the CX3CL1/CX3CR1 axis is dysregulated.

In summary, microglia are key cells in the maintenance of CNS homeostasis. They are essential for the clearance of proteins, such as the extracellular pathological Tau that characterizes tauopathies. As previously mentioned, the amount of phosphorylated Tau (phospho-Tau) at late stages of AD run in parallel with the number of activated microglia. However, a reduction in the phagocytic action of these cells has been shown in such stages. These findings support the notion that during the progression of the disease, there is an uncoupling of microglia activation and phagocytosis that specifically occurs at later stages of AD. Therefore, we propose that this disconnection is one of the mechanisms that drives neurodegeneration in AD and other tauopathies (Figure 2).

## Author Contributions

JP and ML-M contributed equally to this work. MB, JP, ML-M and JÁ drafted the manuscript. All authors read and approved the final manuscript.

## Conflict of Interest Statement

The authors declare that the research was conducted in the absence of any commercial or financial relationships that could be construed as a potential conflict of interest.
